# BiNA: A Visual Analytics Tool for Biological Network Data

**DOI:** 10.1371/journal.pone.0087397

**Published:** 2014-02-13

**Authors:** Andreas Gerasch, Daniel Faber, Jan Küntzer, Peter Niermann, Oliver Kohlbacher, Hans-Peter Lenhof, Michael Kaufmann

**Affiliations:** 1 Algorithmics, Department for Computer Science, University of Tübingen, Tübingen, Germany; 2 Applied Bioinformatics, Center for Bioinformatics, Quantitative Biology Center, and Department for Computer Science, University of Tübingen, Tübingen, Germany; 3 Center for Bioinformatics, Saarland University, Saarbrücken, Germany; 4 Roche Diagnostics GmbH, Pharma Research and Early Development Informatics, Penzberg, Germany; Cincinnati Childrens Hospital Medical Center, United States of America

## Abstract

Interactive visual analysis of biological high-throughput data in the context of the underlying networks is an essential task in modern biomedicine with applications ranging from metabolic engineering to personalized medicine. The complexity and heterogeneity of data sets require flexible software architectures for data analysis. Concise and easily readable graphical representation of data and interactive navigation of large data sets are essential in this context. We present BiNA - the Biological Network Analyzer - a flexible open-source software for analyzing and visualizing biological networks. Highly configurable visualization styles for regulatory and metabolic network data offer sophisticated drawings and intuitive navigation and exploration techniques using hierarchical graph concepts. The generic projection and analysis framework provides powerful functionalities for visual analyses of high-throughput omics data in the context of networks, in particular for the differential analysis and the analysis of time series data. A direct interface to an underlying data warehouse provides fast access to a wide range of semantically integrated biological network databases. A plugin system allows simple customization and integration of new analysis algorithms or visual representations. BiNA is available under the 3-clause BSD license at http://bina.unipax.info/.

## Introduction

Systems Biology aims to study the relationships and interactions between various parts of a biological system and to integrate this information in order to understand its functionality. In this process, the visualization and visual analysis of the network data plays an essential role in understanding complex biological processes and mechanisms. Many tools, which strongly differ in the way of presenting the data, have been developed for visually exploring biological networks [1]. The most popular graphical representations of biological networks are hand-drawn static visualizations, so-called maps. While they are widely in use and appear in countless journal articles and reviews, the representations are static and usually lack interactivity. Typical examples of static visualizations of network data are the Roche Biochemical Pathways Chart [2], the Biochemical Pathway Atlas [3], as well as the KEGG [4] and BioCarta [5] pathway maps.

In addition to the static pathway maps, many tools that dynamically visualize biological networks have been developed in the last decade. Most of these tools have been designed to fulfill the requirements of particular applications.

The stand-alone visualizer Cytoscape [6] is a popular tool offering dynamic network layouts. Its plugin interface makes Cytoscape easy to extend and thus hundreds of third-party plugins are available. It is released under the Lesser General Public License (LGPL) and has a large user and developer base.

Another visualization tool that has been designed specifically for the integrative visual data mining of biological pathway is VisANT [7]. In addition, it allows analyzing network clusters or searching for network motifs. Like Cytoscape, VisANT is freely available and can be extended via its own plugin structure.

VANTED [8] is an open-source workbench that supports the analysis of biological data in a network context. Similar to CellDesigner [9], which has a focus on systems biology modeling, it also supports the network visualization using the Systems Biology Graphical Notation (SBGN) [10].

A problem with visualizing biological data is the size of the networks, which can be very large, e.g., for protein-protein interaction data. OSPREY [11] is a tool that was developed especially for applications that explore these very large networks. Unfortunately, this tool, like many other academic developments, is not maintained any more.

Since many users appreciate the classical layouts used in static biological maps like KEGG, some visualization tools try to dynamically generate similar layouts, which in addition have the advantage of being editable. KGML-ED [12] is such a tool: it reads the KGML files from the KEGG database and visualizes the networks using the KEGG layout information. Apart from the freely available tools, several commercial software solutions exist for the visualization of biological pathways including Elsevier Pathway Studio [13], Ingenuity Pathway Analysis [14], Thomson Reuters Metacore [15], and Cell Illustrator [16].

A review [17] from 2007 and a survey [18] from 2008 compare existing tools for the visual exploration and analysis of biological networks and evaluates their features. In both reviews, the authors state that, although the developments of the last few years have resulted in different tools with various features, the development of applications for the visualization and visual analytics of biological networks still requires further efforts. This observation has been confirmed by a study of Schreiber et al. in [1] from 2009, where seven use cases and corresponding open visualization problems are described. With the advent of high-throughput technologies, it has become more and more important to visualize large-scale omics datasets in the context of networks. The state of the art in this area has recently been reviewed by Gehlenborg et al. [19]. O'Donoghue et al. [20] analyzed the evolution of major software tools in this field regarding the still unmet challenge of true integration and high usability.

A major challenge in visualizing biological networks is that the layouts have to account for additional constraints imposed by biological convention or additional data (e.g., subcellular location). The next step is the visualization of high-throughput data (proteomics, transcriptomics, metabolomics, etc.) in the context of these networks [19]. Here, the amount of data, its incompleteness, its ambiguity, and the diversity of potential data formats are the key problems. In some omics areas standardized data formats are still rather recent developments and their support is thus still scarce [21]–[24].

The aspect of incorporation of external data sources is another important point also addressed by Suderman et al. in [17]. Most of the tools presented can only read data sources in a certain format. Additionally, these tools usually offer preformatted versions of certain public databases. However, for the integration of the user's own data, one must rely on tools for the conversion of data into the required data format, which is in many cases a non-trivial process. Thus, Suderman et al. suggest that all tools should integrate standard file formats, like BioPAX [25], SBML [26], or PSI-MI [27]. A short comparison of these XML formats is also provided by Pavlopoulos et al. [18].

Another major challenge is the improvement of the layouts since most tools do not take into account the underlying biology and drawing conventions known from biochemical textbooks but only the structural relations of the mathematical network. For specific applications, it is however necessary to extend and customize the general layouts to the special needs of the application. Most visualization tools focus on very specific aspects and cannot easily be extended.

Thus, sophisticated layouts for biological networks in general, but also tailored for special applications, need to be developed and combined with existing drawing concepts. Furthermore, the integration of future tasks and applications should be easy. The key challenges in the area of visual analytics of biological networks remain, thus, to combine automated analysis with advanced visualization techniques, and to make the navigation and exploration of huge data sets interactive.

A prior version of the software we present, has been published along with the BN++ data warehouse [28]. BiNA has been re-engineered in its entirety since then. Changes include architectural changes (modularized architecture based on a new plugin model), major changes to the internal data structures, novel algorithms for graph layout, and a completely re-designed graphical user interface.

## Results and Discussion

BiNA is a visual analytics tool for the interactive visualization, exploration, and analysis of biological network data. It uses advanced graph drawing techniques for visualizing the network data. The integrated connection to an R server supports custom pre-analysis of high-throughput (omics) data, which can then be projected onto these networks and visualized and analyzed in a number of ways.

The core of BiNA is a sophisticated visualization concept, combining high-quality graphics, dynamic network visualizations, and very flexible data projections. BiNA provides different network representations and visual styles.

**Figure 1:**
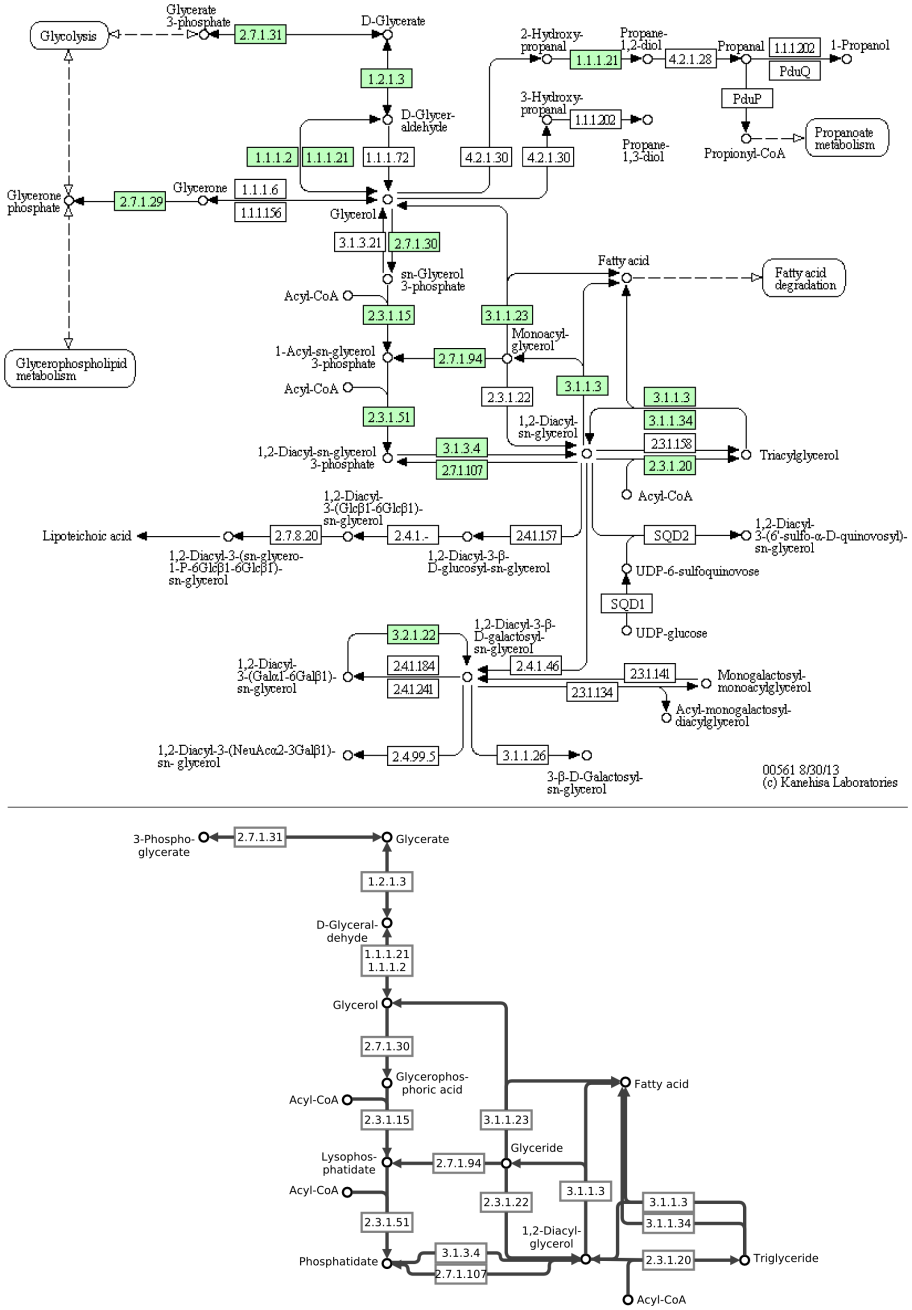
Metabolic pathway representation in BiNA. The KEGG Glycerolipid metabolism of human (on the top) in comparison to the corresponding metabolic representation of BiNA using the KEGG visual style (below). BiNA's KEGG visual style provides layouts of the pathways which are very similar to the KEGG maps. Additionally, BiNA supports filtering of organism-unspecific parts of a pathway, which improves the readability. In this figure, we manually removed disconnected reactions from BiNA's pathway. Furthermore, neighbored pathways can be directly explored and shown in the same visualization, which clearly supports the biological understanding of relationships across borders of canonical pathways (not shown).

Figure 1 illustrates how BiNA visualizes the KEGG Glycerolipid metabolism (bottom) in comparison to the original KEGG pathway (top). Filtering out of pathway components not contained in a given organism improves its readability while maintaining the overall pathway layout. It is also possible to navigate to related pathways by double-clicking on the closed pathway groups nearby.

**Figure 2:**
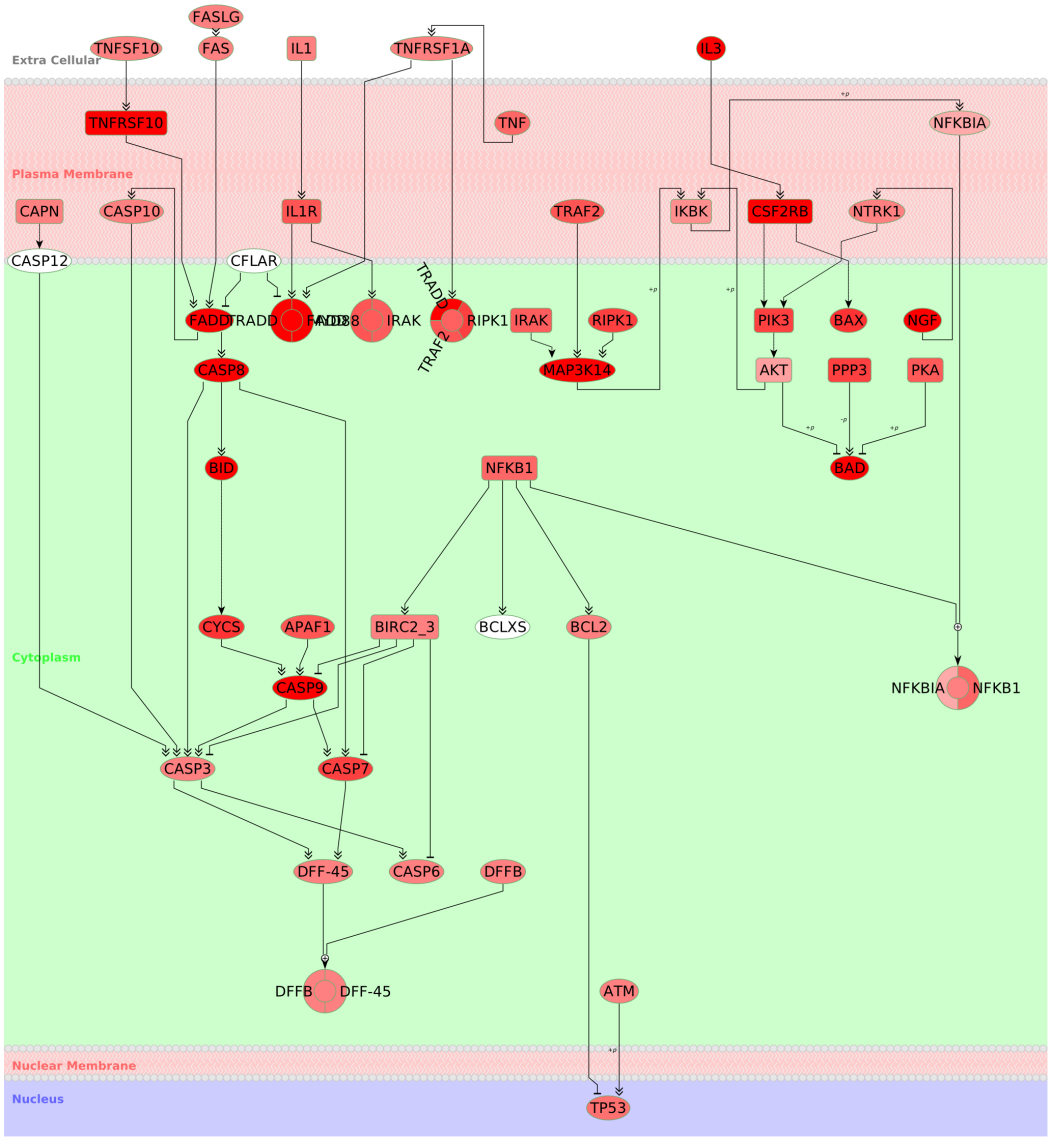
Sub-cellular compartment visualization. The visualization of the KEGG Apoptosis pathway in a layered sub-cellular compartment model demonstrates BiNA possibilities for integrating cellular location information. For this, information, e.g., from SwissProt [29], can be used to assign the proteins to the layout layers, which correspond to an abstract cell model. This representation is meaningful for highlighting signaling cascades into the nucleus. Since, proteins can have multiple cellular locations, it is also possible to validate the compartment assignment by projecting the ambiguity level of the cellular locations to the node colors: From unambiguous (red) via ambiguous (rose) to white (no information available).

In Figure 2, we show a regulatory network containing various proteins, complexes, and families, which are arranged according to sub-cellular location information from the UniProt/SwissProt knowledgebase [29]. BiNA provides a special hierarchical layout algorithm, which supports layer assignment of nodes. The hierarchically organized cellular component part of the gene ontology [30] is used to map location information to the layers in our model. Node attributes (e.g., color) can be used to highlight cases of uncertain or contradictory annotation.

Similar to other tools, BiNA is able to project high-throughput data from various sources to the network. For this, we implemented a generic projection framework, supporting a large number of projection targets. These targets range from simple graph attributes, e.g., color, node size, line thickness, and visibility to complex ones, like sub-cellular compartment association or time series. Time series projections, for example, are realized using small charts, which are drawn instead of node shapes.

**Figure 3:**
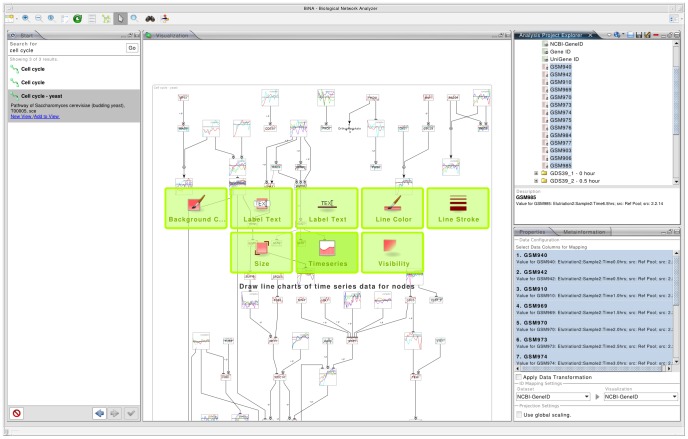
High-throughput data projection in BiNA. High-throughput datasets can be projected onto a network by simple drag and drop operations. The upper right hand side of the view shows available datasets. When one of these datasets is dragged onto the main network visualization, possible network attributes for projection arise (green boxes). Afterwards, a dialog opens and permits a more detailed configuration of the projection (not shown).

The user can load omics data from various file formats, via drag and drop from a spreadsheet program, or online from the gene omnibus database (GEO) [31]. Missing identifiers can be automatically obtained using the UniProt ID mapping service [32]. The data projection itself can be initiated using the menu or via drag and drop by dropping the data onto the desired projection target in a visualization view (see Figure 3).

The imported data can be modified directly in BiNA. It is possible to transform the data based on simple mathematical expression. Access to a number of mathematical functions and operators is provided through an intuitive syntax. User defined functions can be mapped to R expressions [33].

**Figure 4:**
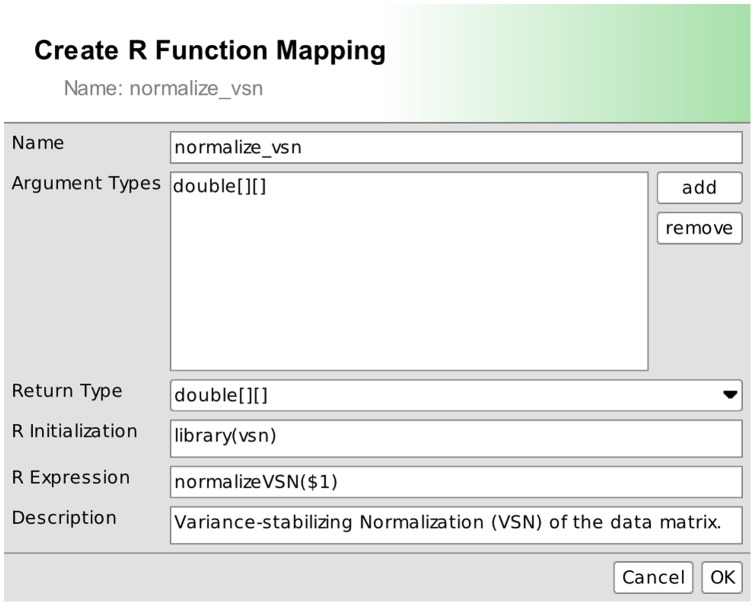
Access R from BiNA. The editor for connecting R expressions with functions in BiNA. The new normalize_vsn(x) function calls the underlying R statement normalizeVSN(x).

Figure 4 illustrates how to implement the well-known variance-stabilizing normalization [34]. The new function *normalize_vsn* takes a matrix as an argument and passes it on to the *normalizeVSN* function of the *vsn* package of R [35].

**Figure 5:**
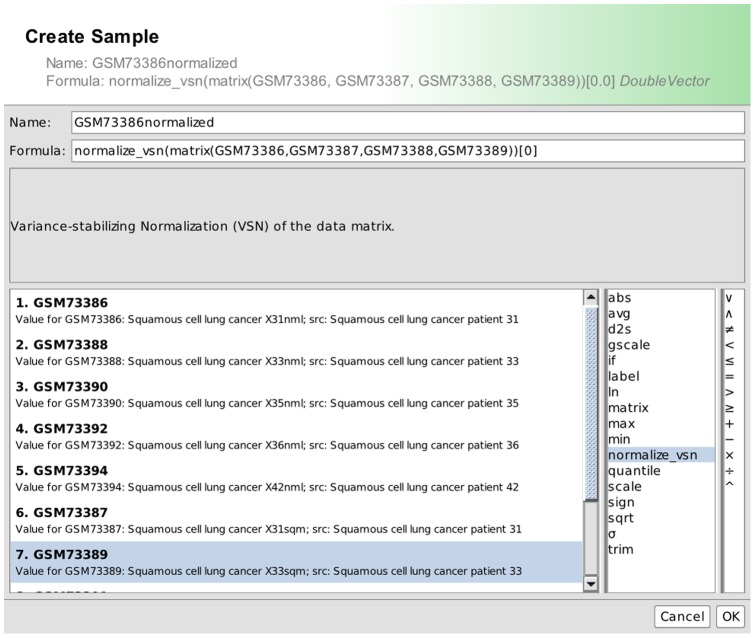
Derive new data sets using R. Creation of a new sample (dataset) using the callback function defined in Figure 4.

The mathematical expression editor (Figure 5) can then call this new function and use it to normalize the input dataset and thus create a new normalized dataset *GSM73386 normalized*. This new dataset can then be stored or mapped onto the network data in BiNA.

For a more complete overview of BiNA's features and a comparison to other tools, we refer to Table S1.

### Applications

**Figure 6:**
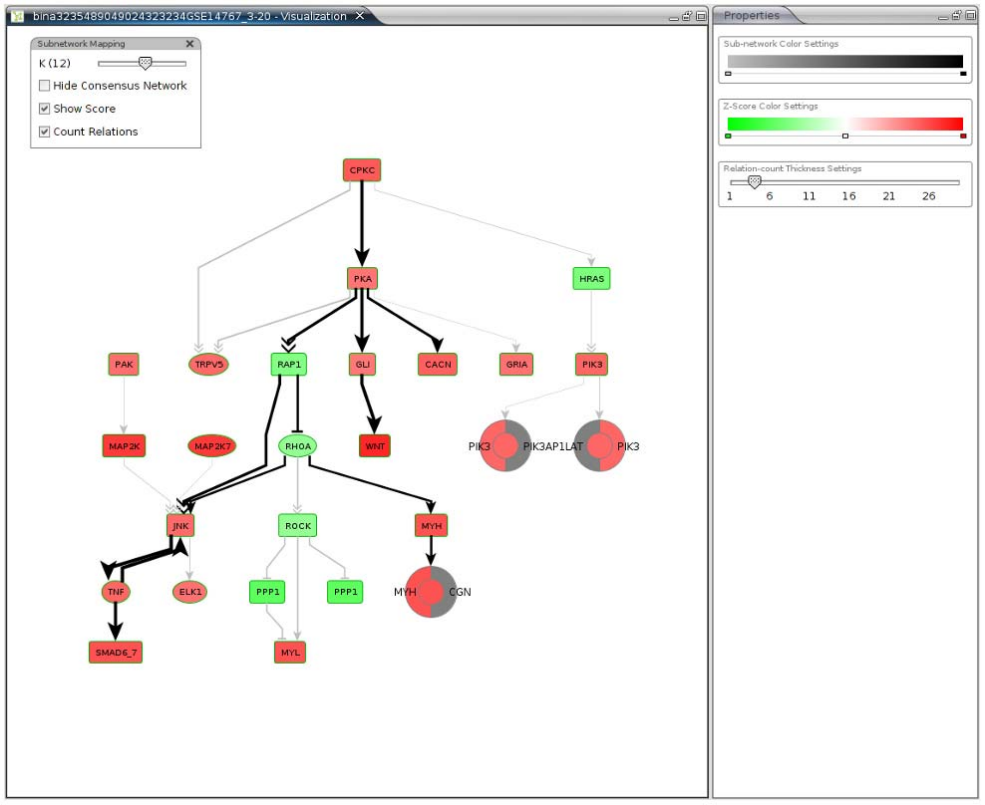
NetworkTrail Application. The results of NetworkTrail can be visualized using BiNA Webstart. The NetworkTrail plug-in of BiNA provides interactive navigation through the found subnetworks using the toolbox in the top-left corner of the visualization, which supports the evaluation of the results. It is easily possible to switch to a certain subnetwork of size k, or to hide the consensus network, which is the union of all found subnetworks. It is also possible to show or hide the score and the number of relations an edge represents. On the right-hand side, the user can adjust some basic visual mapping properties.

BiNA has been used in several application scenarios. For example, we are offering a web service called NetworkTrail [36] for detecting deregulated pathogenic processes that uses BiNA for visualizing the resulting biological subnetworks and pathways. NetworkTrail identifies maximally deregulated subnetworks in directed regulatory networks applying an Integer Linear Programming (ILP) based branch&bound algorithm [37]. Given the degree of deregulation of the genes and proteins that belong to the considered regulatory networks as node scores, the algorithm calculates the heaviest connected subnetwork of a specified size k that contains a designated root node from which all other nodes in the subnetwork are reachable. This root node may represent a molecular key player that might have induced the observed expression changes and hence the pathogenic processes. The web service takes as input a score list of all involved genes or Gene Expression Omnibus (GEO) [38] records that can be used to calculate score lists. NetworkTrail calculates all maximally deregulated subnetworks in a user-specified range and enables the user to visualize the results using either the Java Webstart-based BiNA or Cytoscape Web [39]. In Figure 6, the colors of the nodes represent the degree of up- and down-regulation of the corresponding genes and the slider enables the user to scan the different subnetworks easily. Moreover, BiNA “allows for a complete customization of the visualization, including layout, colors and node style” [36]. Especially, the hierarchic layout offered by BiNA facilitates the interpretation of the detected signaling cascades showing often the information flow from receptors on the cell membrane to transcription factors in the nucleus.

Other applications where we successfully used BiNA for visually analyzing the algorithmic results are miRtrail [40], FIDEPA [41], GeneTrail Express [42], and GeneTrail [43].

## Design and Implementation

### Basic Architecture

**Figure 7:**
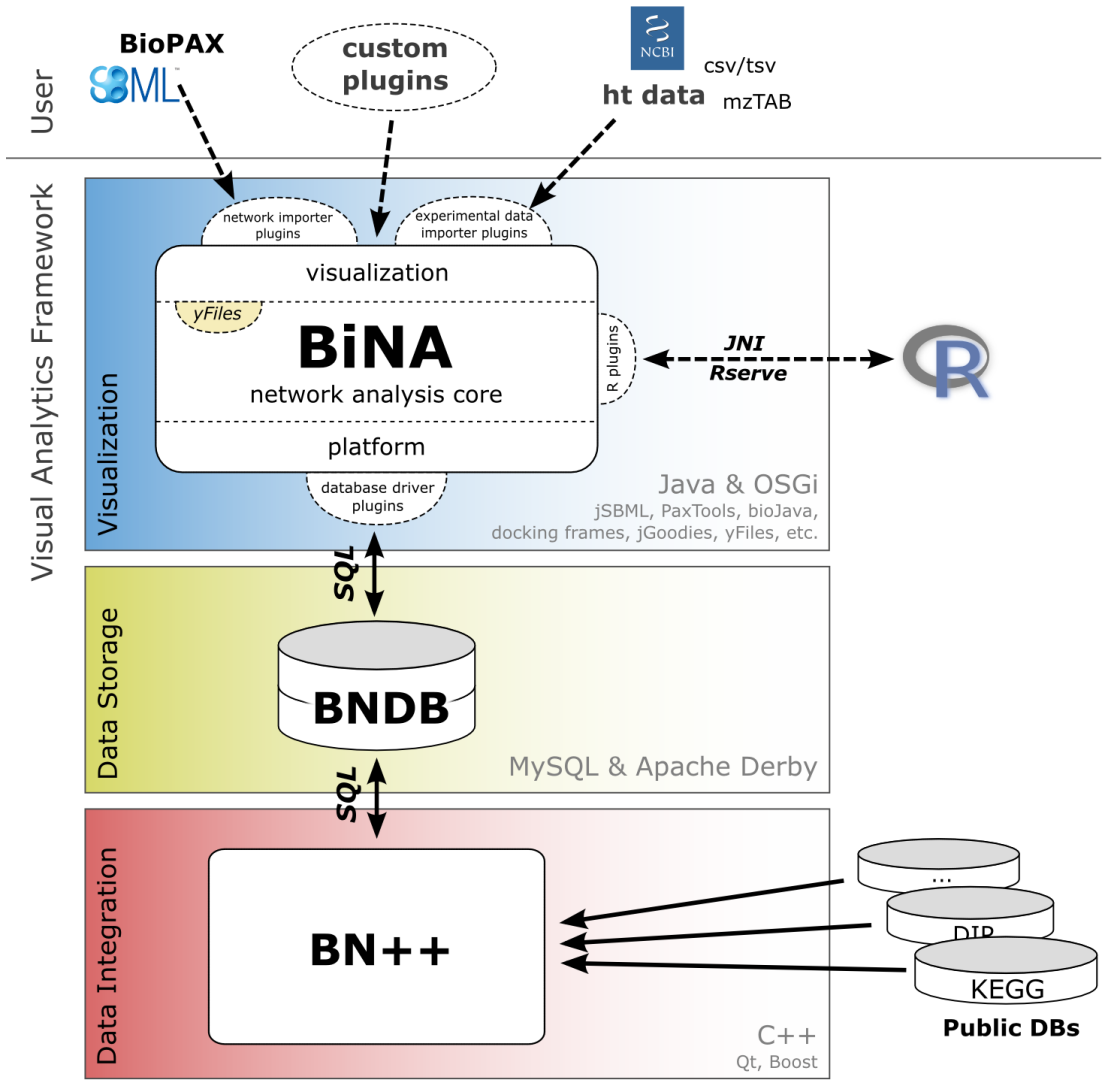
Architecture sketch of BiNA and BN++. BiNA acts as a visualizer for the BN++ data warehouse system, which semantically integrates several biological databases and stores them into BNDB. BiNA is able to access BNDB directly via SQL, either the MySQL or the Apache Derby version. BiNA consists of a number of plug-ins (OSGi bundles), which are packed together for distribution. Using these plug-ins BiNA can import various file formats, use an R server for processing experimental data, and visualize and analyze networks in different contexts. The user is able to extend the functionality of BiNA using the public API of the OSGi bundles.

In Figure 7, we show the architecture of BiNA and its connection to the BN++ data warehouse [28]. BN++ is a data warehouse system, which can import various biological databases, e.g. KEGG, DIP, UniProt. These databases have been semantically integrated into the BioCore model, which is then mapped to a relational database system (BNDB [44]). By default, this is a MySQL or Oracle database, but we also provide conversions to an Apache Derby database, for environments where a database server is not available. BiNA is able to access BNDB directly, either the MySQL version or the Apache Derby version.

BiNA itself is a modular software tool built upon OSGi [45] and consists of a large set of core bundles (OSGi synonym for module or plugin) providing all the functionalities described in this paper.

Each core bundle provides a API, which can be used by software developers to extend the functionality of BiNA. These bundles usually correspond to certain application scenarios, e.g., for using BiNA Webstart in an online workflow for visualizing results in a customized way. The developer documentation is available at http://bina.unipax.info/development and contains JavaDocs and a comprehensive example plugin, which addresses different use cases.

The underlying OSGi 4.3 platform provides dynamic loading and unloading of bundles during runtime, automatic handling of native libraries, and a full-featured component/bundle system, which becomes more and more necessary in large software projects. The main application window (GUI) of BiNA is divided into several dynamic views using the Docking Frames window management library [46]. It provides many features (e.g., collapsed, detached, and full screen mode) for rearranging views inside the main window. Well-arranged working environments can be pre-defined using, so-called, perspectives. We extended the library by content- and focus-sensitive menu items, which simplifies the access to currently important functionality.

BiNA distinguishes between views and editors. While a view exists only once, editors can have multiple instances, e.g., for different visualizations.

### Network Visualization Concepts

The core of the visualization is a sophisticated visualization model. From a set of universal base components, different visualization styles are realized, e.g., for metabolic and regulatory networks. The latter ones include gene regulatory, signaling, and protein-protein interaction networks.

Base components of the visualization model like nodes, edges, groups, hyperedges, and stars combine the simplicity of graphs with the readability of hierarchically structured elements. During the visualization process these components will be translated into a hierarchic graph using the graph library interface of BiNA. An implementation of this interface is provided by the separate yFiles graph library [47] bundle, which is free for usage in combination with BiNA, but not available for open source (see section Availability).

Groups of the visualization model are realized using hierarchic nodes and can be opened and collapsed again using mouse gestures. They can contain their own graph layout, which supports multiple layouts in one visualization view and can be used for, e.g., laying out two biological pathways in a different way nearby.

Every component has an additional graphical abstraction layer describing its visual appearance and available user interactions. This layer can be exchanged by a different visual style, for example, the KEGG visual style for the metabolic representation.

Since groups can be collapsed and single components can be made invisible, single changes to the model can result in a large number of changes to the resulting graph. We meet this, by only updating those parts of the graph, which have been marked as modified by the changes to the model. We further distinguish between topological, visual, and layout changes to avoid unnecessary calculations, which heavily accelerate the rendering process in many cases.

This is also the reason why we subdivide the rendering process into three steps: (1) the construction of the graph topology, (2) assignment of graphical attributes (e.g., color and shape) to each node and edge, and (3) laying out the graphical elements on the screen.

Data projection (see section below) can influence each of these steps. For example, the projection of omics data to node visibility can filter some nodes in the first step. The subdivision into these steps now improves the rendering speed, since not all nodes and edges have to be processed by step two and three, which becomes important for large networks. Figure S1 demonstrates the celerity and linear scalability of rendering up to 100,000 protein-protein interactions with BiNA.

Every component of the visualization model has a set of properties, e.g., label text, shape, size, text font, color, line width. These properties have default values that can be changed globally with a predefined visual style. These properties can, however, be overridden by the user at any time. The resulting graphical representation can thus be customized in any way the user desires.

The network visualizations can be saved using the BiNA specific file format (BML). The saved visualization contains also all data sets projected to the visualization and the visualization-wide properties.

### Network Data Access

As discussed above, BiNA is able to directly access biological networks from a BNDB [44] database (see Figure 7). The database can easily be searched and visualized using tasks available in the Start view. Of course, BiNA is able to import networks given in BioPAX, SBML, and SIF format. BioPAX and SBML files can be directly visualized by selecting an appropriate visualization style. Similar to the BNDB, they can be used as an in-memory database, providing the same functionality. Using the SIF format, the user can select how to translate certain relation types contained in a SIF file. Available translations are, for example, complex building, metabolic co-factor of a reaction, phosphorylation, activation, or inhibition. The SIF importer is also able to automatically map identifiers found in a SIF file to proteins or compounds found in the BNDB database. This gives fast access to metadata and allows network explorations beyond information given by the loaded file.

### Data Projection

A generic projection framework supports the mapping of arbitrary external information, primarily omics data sets, onto the network data. As noted before, data projection can influence a large number of visualization attributes, like color, size, stroke, and more complex ones like reaction direction, or subcellular location. The projected data can be imported from suitable files (e.g., mzTab, comma separated values format (CSV/TSV), SOFT), dragged & dropped from text/spreadsheet editors, or downloaded from the GEO database [31]. Afterwards, the data is available in the *Analysis Project Explorer* view for processing and projection onto network visualization.

The data itself is organized in a tabular fashion. Data columns can be of different types. Rows correspond to individual components of the network (i.e., proteins, genes, metabolites) and are identified by a single identifier or a set of identifiers (typically database accession IDs). Columns can be grouped in order to express sample semantics (e.g., replicate structure). Identifiers will be mapped to the internal identifiers of the network nodes. If necessary, external identifier mapping services (UniProt) are employed to translate between different identifier systems.

BiNA will use the selected sample of the series for data projection. If multiple samples are selected, BiNA will automatically try to compute a temporary sample consisting of their mean values, which will then be used for projection. It is also possible to enter a custom formula using an intuitive syntax to calculate the temporary sample. The user can choose from a large set of built-in functions or provide new user-defined functions, which will be passed on to and evaluated by R.

In the latter case, BiNA must be connected via Java Native Interface (JNI) to an R installation or via network to a server running the Rserve package. The connection to R also enables loading of arbitrary matrix data from R into a series in BiNA and vice versa, such that existing workflows in R can be reused. Derived samples can be exported again together with the series, either in the BiNA data format (BDF) or in comma/tab separated values format (CSV). If BiNA is connected to R using JNI, the *R Console* view is available for the user, which grants full callback access to the underlying R session.

### Network Analysis

Since, the network visualization of BiNA uses the concept of hierarchic graphs, it is not reasonable to apply standard graph algorithms on the resulting visualization graph, for example, a shortest paths search. Especially, the groupings of protein families, protein complexes, biochemical reactions, and pathways, and the multiple representation of entities used for visualization aspects, can conflict with graph theoretic interpretations. To meet this problem, the different network representations of BiNA provide a configurable way of flattening the visualization graph. For example, the hierarchical modeling of protein families can be flattened by splitting the node representing the family into its individual members. This feature can be used by developers to access the underlying biological network of BiNA. It is also used for exporting visualizations into simple graph formats (e.g., GraphML).

BiNA provides also a way to project the results, calculated on the simple graph, back to the visualization. We provide two example algorithms, a *k* shortest paths and a breadth-first search. The resulting paths and distances can be highlighted in the visualization using node and edge colorings.

### Metabolic Network Representation

The default visual style of the metabolic network representation provides drawings known from biochemical textbooks. For this, reaction arrows are drawn using quadratic curves and instead of node shapes, the chemical structure of metabolites can be shown by painting MOL files from the KEGG database. The resulting network is a bi-partite graph, containing reaction nodes (enzymes) and metabolite nodes. As noted before, reactions are realized using groups containing the co-factors of a reaction. Since the reaction groups do not have their own layout style, co-factors and their edges are integrated into the parental layout, if the group is opened.

The KEGG visual style is built upon the default one. It provides different properties for the visualization elements, and uses a special KEGG layout algorithm for pathways to imitate the KEGG maps. The layout information is provided in parts by the KGML files of KEGG. For example, a KGML file contains information about node positions, but lacks information about edge routes, thus, they must be computed by the layouter.

The metabolic representation provides functionality for improving the readability and clarity of the visualization, like context-specific hiding of co-factors and aliasing of compounds and reactions. Several strategies for selecting co-factors are provided to the user, e.g., by information from the source data, editable pre-defined lists, heuristics, or manually. Furthermore, the integration of context-specific pathway validation strategies (e.g., those presented in [48], [49]) is planned.

### Regulatory Network Representation

The regulatory network representation visualizes various regulative interactions and complex assemblies of entities (proteins and metabolites). These can be organized in complexes and families, which are realized using groups. Similar to the metabolic representation, the regulatory one supports aliasing of entities. This improves the readability, if multiple pathways, which are also realized as groups, contain the same entity. Different instances of an entity can be highlighted and it is possible to merge instances again. Another feature is the exploration of the underlying data source. For this, the user can explore the neighborhood of an entity, either upstream, downstream, or both, by adding these regulative interactions to the visualization.

### Graph Layout

For rendering and layout algorithms, BiNA uses the yFiles for Java graph library. We provide the *yFiles* bundle for BiNA, which implements the graph library developer interface of the *Network Analysis* bundle. Using this interface, BiNA is able to access yFiles features indirectly, with respect to the 3-clause BSD. This bundle also provides a number of layout algorithms given by the yFiles library. Most notable for our purposes are the yFiles hierarchic layouter, the organic (forced-directed) layouter, and the orthogonal layouter. These layout algorithms have been extended to our specific needs, supporting the layout criteria of the metabolic and regulatory network representations, and their visual styles.

In the metabolic representation, for example, the organic layouter is extended to support the readability by laying out dangling co-factors according to the reaction flow. For the regulatory representation, which supports changing the visualization background to visualize subcellular locations, we extended the hierarchic layouter of the yFiles graph library to support layer assignments in an easy way.

## Conclusion

BiNA, the Biological Network Analyzer, is a mature tool for the integrative analysis of omics data in a network context. In contrast to other tools, it permits not only the direct import of networks and omics data from flat files, but also features a direct connection to an integrated data warehouse. By supporting common file formats for transcriptomics, metabolomics, and proteomics as well as arbitrary tabular data, nearly any data set of interest can be projected onto the networks and explored interactively. A generic projection system supports users in creating visually concise and information-rich visualizations that can be directly exported in publication quality. The interface to the statistical programming language R furthermore enables a wealth of existing statistical methods. These can be easily applied to the datasets loaded in BiNA and results of the statistical analysis can be easily projected back onto the network visualization.

The integration of advanced graph drawing techniques permits a pleasing graphical representation of the networks. Inclusion of well-established layouts (e.g., the KEGG layouts) provides easily recognizable pathways while adding the benefits of interactive navigation and simultaneous display of multiple pathways. Where these layouts do not suffice, the built-in editing capabilities permit extension, correction, and curation of the representation as well as the underlying data.

BiNA's standardized plugin interface permits developers to add custom extensions whenever needed. We expect that additional plugins will extend the functionality soon.

### Availability

BiNA (version 2.4.1) is available free of charge under an open-source license (3-clause BSD license) with exception of the yFiles bundle, which is free but not open-source. Source code, documentation, and installable packages are available from the project web site at http://bina.unipax.info/ and Sourceforge at http://sf.net/p/bina/. Since BiNA is written in Java, it runs on every platform supported by Oracle's JRE 7 or OpenJDK 7. We provide prepackaged versions for Windows, Mac OS X, and Linux containing an executable for starting BiNA. In addition, we provide a Java Webstart version at http://webstart.bina.unipax.info/2.4.1/.

## Supporting Information

Figure S1 **Rendering time of protein-protein interaction networks of different sizes in BiNA.** BiNA allows loading of networks having more than 100,000 edges using a standard desktop PC (Linux 64bit, Quad Core Intel CPU Q9400@2,66GHz, 8 GB RAM). In the Figure we give some testing results for rendering Protein-Protein Interaction networks from a SIF file containing 50 to 100,000 edges (interactions) using the organic layouter. The overall rendering time for 10,000 interactions took about 4.2 seconds and for 100,000 interactions about 53 seconds. Note that depending on the network size, we dynamically change the layout settings, to improve its quality in smaller (human readable) networks.
(DOCX)

Table S1 **Comparison of major functionalities between BiNA, Cytoscape, VANTED, and CellDesigner.** The table shows a comparison of major functionalities between BiNA, Cytoscape, VANTED, and CellDesigner. Since all tools are extendable by plug-ins, we compare the distributed versions and mark features fulfilled by an additional plugin with an asterisk. Major differences of the tools can be found in the data access methods, where the functionality of BiNA is heavily affected by the presence of the BN++ data warehouse, which provides access to a large number of integrated databases. Another essential difference is BiNA's network visualization model, which is based on hierarchical graphs and focuses comprehensive, dynamic, and interactive visualizations for metabolic, regulatory, and signaling networks of high quality. Although, CellDesigner has also a hierarchical visualization model supporting hierarchical groupings, it is more focused on metabolic network simulation and lacks explicit functionality for regulatory and signaling network visualization.
(DOCX)
