# Supplementary material for: BiNA: A Visual Analytics Tool for Biological Network Data
Source: PLoS One. 2014 Feb 13;9(2):e87397. doi: 10.1371/journal.pone.0087397 (PMC3923765; doi:10.1371/journal.pone.0087397)
Supplement: Figure S1 — Rendering time of protein-protein interaction networks of different sizes in BiNA. BiNA allows loading of networks having more than 100,000 edges using a standard desktop PC (Linux 64bit, Quad Core Intel CPU Q9400@2,66GHz, 8 GB RAM). In the Figure we give some testing results for rendering Protein-Protein Interaction networks from a SIF file containing 50 to 100,000 edges (interactions) using the organic layouter. The overall rendering time for 10,000 interactions took about 4.2 seconds and for 100,000 interactions about 53 seconds. Note that depending on the network size, we dynamically change the layout settings, to improve its quality in smaller (human readable) networks. (DOCX) [file pone.0087397.s001.docx]

Figure S1: Rendering time of protein-protein interaction networks of different sizes in BiNA.
