# Supplementary material for: BiNA: A Visual Analytics Tool for Biological Network Data
Source: PLoS One. 2014 Feb 13;9(2):e87397. doi: 10.1371/journal.pone.0087397 (PMC3923765; doi:10.1371/journal.pone.0087397)
Supplement: Table S1 — Comparison of major functionalities between BiNA, Cytoscape, VANTED, and CellDesigner. The table shows a comparison of major functionalities between BiNA, Cytoscape, VANTED, and CellDesigner. Since all tools are extendable by plug-ins, we compare the distributed versions and mark features fulfilled by an additional plugin with an asterisk. Major differences of the tools can be found in the data access methods, where the functionality of BiNA is heavily affected by the presence of the BN++ data warehouse, which provides access to a large number of integrated databases. Another essential difference is BiNA's network visualization model, which is based on hierarchical graphs and focuses comprehensive, dynamic, and interactive visualizations for metabolic, regulatory, and signaling networks of high quality. Although, CellDesigner has also a hierarchical visualization model supporting hierarchical groupings, it is more focused on metabolic network simulation and lacks explicit functionality for regulatory and signaling network visualization. (DOCX) [file pone.0087397.s002.docx]

Table S1: Comparison of major functionalities between BiNA, Cytoscape, VANTED, and CellDesigner.

|  | | **BiNA** | **Cytoscape** | **VANTED** | **CellDesigner** |
| --- | --- | --- | --- | --- | --- |
| **General** | | | | | |
|  | Version | 2.4.1 | 3.0.2 | 2.1.0 | 4.3 |
|  | Released | 2013-12-06 | 2013-07-19 | 2012-08-07 | 2013-02-01 |
|  | License | BSD 3-clause | LGPL2.1 | GPL2.0 | Freeware |
|  | Execution Environment | Java 7+ | Java 6+ | Java 6+ | Java 6+ |
|  | Distribution | stand-alone, Java Webstart | stand-alone | stand-alone, Java Webstart | stand-alone |
|  | Support for plugins | yes, OSGi | yes, OSGi | yes | yes |
| **Data Access** | | | | | |
|  | Data Warehousing | BN++ | - | - | - |
|  | Online Pathway Databases | integrated into BN++ | Pathway Commons and PSICQUIC Web Services | MetaCrop, RIMAS, KEGG^1)^ | BioModels, PantherDB, JWS Online, SABIO-RK |
|  | Online HT Databases | GEO database | * | - | - |
| **Biological Exchange Formats** | | | | | |
|  | BioPAX | in | in/out | in/out | - |
|  | SBML | in | in/out | in/out | in/out |
|  | PSI-MI | - | in/out | - | - |
|  | SIF | in/out | in/out | in | - |
|  | KGML | in (via BN++) | * | in | - |
| **Network Visualization** | | | | | |
|  | Visualization Model | hierarchic graph | graph (*) | graph | hierarchic graph |
|  | Automated Layout | own, enhanced yFiles | own, yFiles, JGraph, prefuse | own, Graphviz | yFiles |
|  | Editable & manual Layout | yes | yes | yes | yes |
|  | Hierarchical Grouping | yes | not shown, * | - | - |
|  | Layout per Group | yes | - | - | - |
|  | Data Exploration | yes | * | - | - |
|  | Network Representation Styles | metabolic, regulatory, generic graphs, * | generic graphs, * | metabolic, generic graphs | generic graphs |
|  | Visual Styles | configurable, KEGG, cellular compartments | configurable, * | configurable, SBGN | configurable |
|  | Cellular Compartments | regulatory networks | * | - | metabolic networks |
| **Data Mapping** | | | | | |
|  | Multiple Omics Levels | yes | yes | yes | - |
|  | Time series | yes | * | yes | - |
| **Analysis** | | | | | |
|  | Graph algorithms | yes | * | yes | - |
|  | Clustering | - | * | yes | - |
|  | Correlation | - | * | yes | - |
|  | General statistics | - | * | yes | - |
|  | * additional plugin(s) required, ^1)^ if user has license | | | | |
